# The Metagenomic Telescope

**DOI:** 10.1371/journal.pone.0101605

**Published:** 2014-07-23

**Authors:** Balázs Szalkai, Ildikó Scheer, Kinga Nagy, Beáta G. Vértessy, Vince Grolmusz

**Affiliations:** 1 PIT Bioinformatics Group, Eötvös University, Budapest, Hungary; 2 Laboratory of Genome Metabolism, Institute of Enzymology, Research Center for Natural Sciences, Hungarian Academy of Sciences, Budapest, Hungary; 3 Department of Applied Biotechnology and Food Sciences, Budapest University of Technology and Economics, Budapest, Hungary; 4 Uratim Ltd., Budapest, Hungary; Chang Gung University, Taiwan

## Abstract

Next generation sequencing technologies led to the discovery of numerous new microbe species in diverse environmental samples. Some of the new species contain genes never encountered before. Some of these genes encode proteins with novel functions, and some of these genes encode proteins that perform some well-known function in a novel way. A tool, named the Metagenomic Telescope, is described here that applies artificial intelligence methods, and seems to be capable of identifying new protein functions even in the well-studied model organisms. As a proof-of-principle demonstration of the Metagenomic Telescope, we considered DNA repair enzymes in the present work. First we identified proteins in DNA repair in well–known organisms (i.e., proteins in base excision repair, nucleotide excision repair, mismatch repair and DNA break repair); next we applied multiple alignments and then built hidden Markov profiles for each protein separately, across well–researched organisms; next, using public depositories of metagenomes, originating from extreme environments, we identified DNA repair genes in the samples. While the phylogenetic classification of the metagenomic samples are not typically available, we hypothesized that some very special DNA repair strategies need to be applied in bacteria and Archaea living in those extreme circumstances. It is a difficult task to evaluate the results obtained from mostly unknown species; therefore we applied again the hidden Markov profiling: for the identified DNA repair genes in the extreme metagenomes, we prepared new hidden Markov profiles (for each genes separately, subsequent to a cluster analysis); and we searched for similarities to those profiles in model organisms. We have found well known DNA repair proteins, numerous proteins with unknown functions, and also proteins with known, but different functions in the model organisms.

## Introduction

The vast field of computer science, termed artificial intelligence (AI), offers powerful methods for distilling relevant information from large sets of data. Metagenomic databases have been increasingly used in the recent years to investigate the bacterial composition of samples taken from a variety of environments. To analyze and compare different genomic data, Hidden Markov Models [1] provide a useful methodology.

A Hidden Markov Model, applied to protein sequences, is basically a random amino acid sequence generator with multiple internal states, two of which are distinguished as START and STOP states. The generator starts from the START state. Until it arrives to the STOP state, it repeats the following two steps:

- it outputs a random amino acid, then
- it moves to a random new state (typically not in uniform distribution).

The role of the multiple internal states is that the probability distribution of the output amino acid and the distribution of the new state both depend on the current state. The model is named “hidden” because the internal states cannot be unambiguously determined by observing the output sequence.

HMMs are particularly useful because they can be trained by a set of input sequences to output similar sequences: if we have proteins of related functions, then we can build a Hidden Markov Model which will generate random amino acid sequences as output, similar to the ones used in training. It is even a more useful property of HMMs that if we take any amino acid sequence, denoted by *w*, our model can easily tell us the probability of generating exactly that sequence *w* as an output.

Consequently, if we have a HMM trained on a certain set of proteins, then the same HMM can assign higher scores (i.e., probabilities) to proteins *similar* to the training set, and lower scores (i.e., probabilities) to proteins *dissimilar* to the training set. Note that this scoring is usually not homogeneous as in the case of BLAST [2] and its clones [3]: in HMM models conservative subsequences are differentiated from those appearing in variable regions.

In the present work, we have applied HMM in a novel way to suggest and possibly discover still unknown protein functions in several well-studied model organisms. Starting from sequence alignments for proteins involved in DNA damage repair, we created Hidden Markov Models and used these models to search for similar genes in the metagenomic samples from different environments. Combining the original HMM with the genes found in the metagenomes, we created a second, more trained HMM that we used to interrogate proteomes of higher order model organisms. This search (termed as “Metagenomic Telescope” in the present study) generated numerous novel hits in the higher order organisms, containing proteins previously not yet described as closely similar to the DNA damage repair proteins. These results indicate the Metagenomic Telescope may be a powerful method for the identification of novel proteins in higher order model organisms.

## Methods

**Figure 1:**
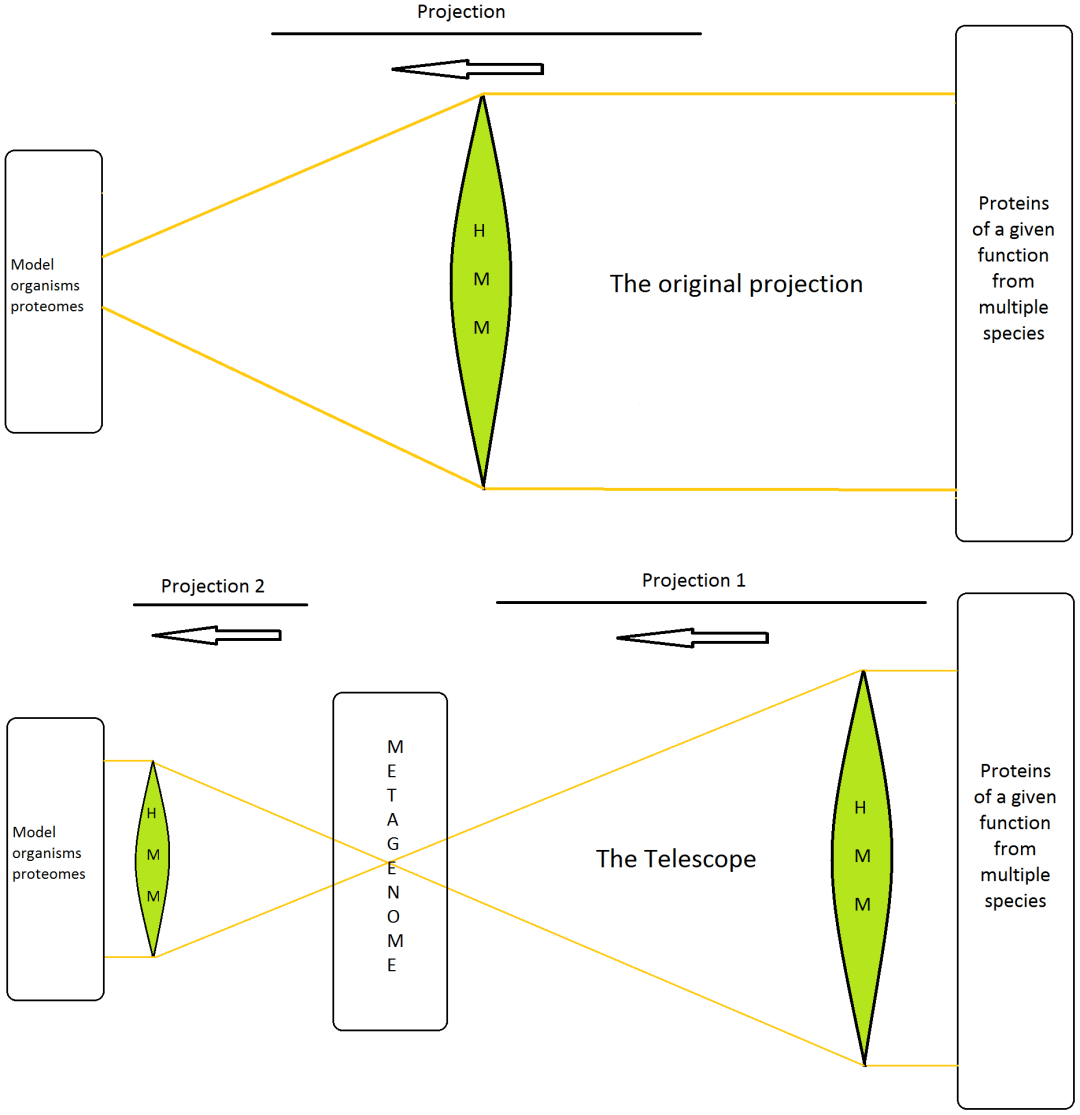
The original projection (upper panel) compared to the scheme of the Metagenomic Telescope (lower panel). Projection 1 discovers genes or proteins in the metagenome that probably have similar function as the well-known starting proteins in front of the objective lens on the right hand side. Projection 2 directly identifies these proteins within the proteomes of the model organisms (as a set of UniProt accession numbers).

First, we took some known *E. coli* and Archaean occurrences of a specific enzyme as listed in Table 1. We aligned these similar proteins using Clustal Omega [4]. The aligned sequences were then used to train a HMM with the hmmbuild utility of the HMMER3 package [5]. We term the resulting model as the “original HMM” (cf. Figure 1).

**Table 1:** Protein families and proteomes used in the present study.

Protein families	Archaea proteomes in “original HMM”	Eukaryotic proteomes **screened by HMM**
dUTPase	Aeropyrum pernix	Saccharomyces cerevisae
uracil-DNA glycosylase (UNG)	Archeoglobus fulgidus	Arabidopsis thaliana
thymine-DNA glycosylase (TDG)	Halobacterium salinarum	Caenorhabditis elegans
Archaeal UDG	Haloferax volcanii	Drosophila melanogaster
NTHL1	Methanobacterium thermoautotrophicum	Danio rerio
OGG1	Methanococcus jannaschii	Gallus gallus
Rad50	Methanococcus maripaludis	Bos taurus
Mre11	Methanosarcina acetivorans	Canis lupus
	Pyrococcus abyssi	Mus musculus
	Pyrococcus furiosus	Sus scrofa
	Pyrococcus horikoshii	Rattus norvegicus
	Sulfolobus acidocaldarius	Homo sapiens
	Sulfolobus islandicus	
	Sulfolobus solfataricus	
	Thermococcus kodakaraensis	

This “original HMM” was used twice: once in the direct projection to the model organisms (producing “original hits”), and second time for Projection 1 in the Telescope (here it represents the first step for producing “telescopic hits”).

In the original projection, similarity scores are assigned to the protein sequences of the model organisms: the output of this single projection is the set of the highest scored proteins, using an inclusion threshold of E-value ≤10^−6^, found in the proteomes of the model organisms. We termed these highest scored proteins as “original hits” (this step is visualized on the upper panel of Figure 1).

For the application in the Telescope, we first extracted open reading frames from the metagenomes with the getorf application of EMBOSS [6], then applied the hmmsearch utility of HMMER3 [5] on the “original HMM” and the database of amino acid sequences extracted from each metagenome. The result of this search consists of hits in the metagenome and are referred to as “metagenome matches”.

Three extreme metagenomes in the present study were accessed through the CAMERA portal [7].

### Richmond Mine in Iron Mountain

CAMERA accession code: CAM_PROJ_AcidMine. The Iron Mountain, California mine was closed in the sixties. Later, the large, underground pyrite depositories became exposed to atmospheric oxygen and moisture, producing one of the most acidic mine drainages on Earth [8]. The metagenome consists of the data gained by sequencing samples from the thick, pink biofilm in this acidic and hot (42°C) environment, containing iron-oxidizing bacteria and other species.

### Yellowstone Bison hot spring

CAMERA accession code: CAM_PROJ_BisonMetagenome. The Bison Pool environment is an alkaline hot spring in the Sentinel Meadow of Yellowstone National Park, situated in Wyoming, U.S. The samples were collected from sites with water temperature of 56°C through 92°C [9]–[11].

### Phosphorus removing (EBPR) sludge community

CAMERA accession code: CAM_PROJ_EBPRSludge. The samples were taken from an enhanced biological phosphorus removal (EBPR) sludge community from the Thornside Sewage Treatment Plant in Brisbane, Queensland, Australia.

**Figure 2:**
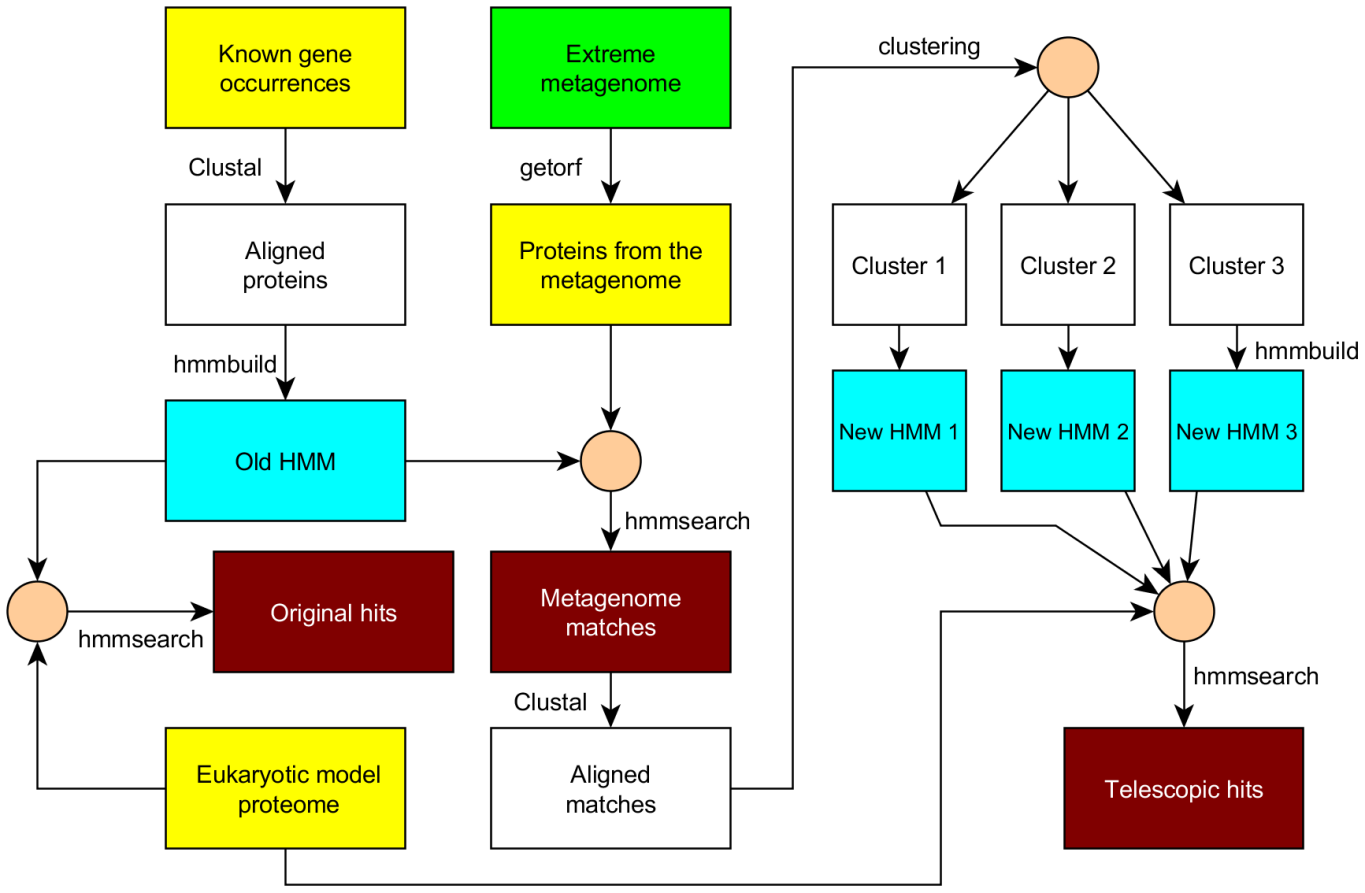
The flowchart of the Metagenomic Telescope applied to DNA repair enzymes.

The metagenome matches (cf. Figure 1) were aligned and clustered using the OPTICS method [12]. The clusters were then used as inputs of hmmbuild [5], which yielded the “new HMMs”. In other words, these models have been built on possible unknown DNA repair enzymes found in the metagenome. We then performed the final step in the process pipeline, i.e., testing both the original and the new, telescopic HMMs on the proteomes of higher level organisms. As visualized on Figure 1, we compared the results of the projection on the upper panel and the projections of the lower panel. These organisms included *Arabidopsis thaliana, C. elegans* and *E. coli* as well as mouse, rat, human, and other model species. The flowchart of the application of the Metagenomic Telescope is shown on Figure 2. After the last projection (Projection 2 on Figure 1), the highest scoring proteins were selected, using again an inclusion threshold of E-value ≤10^−6^. These proteins were termed as “telescopic hits”.

Our goal was to examine whether the possible new DNA repair enzymes found in the metagenomes could be used for finding new DNA repair enzymes in the model organisms as well. This included comparison of the results of the searches with the original and the new models, respectively.

## Results and Discussion

### Design of the Metagenomic Telescope

The optical (refractive) telescope applies two projections: the first projection is done by the objective lens, the second by another lens called “the eyepiece”: through the eyepiece one can see the enlarged image, generated by the objective.

Our Metagenomic Telescope also consists of two projections, each one is performed by applying HMMs. The key point is making use of *metagenomes* in the projections: *first* we project *to* metagenomes, then we project *from* metagenomes. The lower panel of Figure 1 describes these two projections, together producing the “telescopic hits”; and compares these to a single HMM projection on the upper panel of Figure 1, producing the “original hits”.

The starting point is a set of proteins of similar function or structure, taken from well–annotated organisms. This set is the teaching set for both the first HMM in the Metagenomic Telescope and the single HMM of the original projection.

In the original HMM or the original projection (upper panel of Figure 1), we use the HMM constructed in the step for finding similar protein sequences in model organisms: this is the only projection we use here. Using that HMM, similarity scores are assigned to the protein sequences of the model organisms. The output of this projection is the set of the highest scored proteins found in the proteomes of the model organisms (termed as “original hits”).

In contrast, in the Metagenomic Telescope (lower panel of Figure 1), we apply two projections:

**Projection 1 in the Telescope.** Here we use the same HMM as in the original projection, but now we search for high-scored protein sequences in the metagenomes instead of proteins in the model organisms.

**Projection 2 in the Telescope.** The starting point is the highest scored proteins from the metagenome. After a suitable clustering, a new – second – HMM is built: its teaching set consists of these high scored proteins. Next, the proteomes of some model organisms are considered, and by this second HMM, similarity scores are assigned to the protein sequences of the model organisms. The output of the second projection is the set of the highest scored proteins found in the proteomes of the model organisms (termed as “telescopic hits”).

We believe that our telescope will facilitate the identification and annotation the functions of proteins in model organisms, since the diversity of well-chosen metagenomes is expected to help to assign new, still unknown functions to a number of proteins.

### Proof of Concept: DNA Repair Enzymes

As a proof of concept, we applied the Metagenomic Telescope to DNA repair enzymes as the starting set of proteins, and metagenomes, found in extreme environments (acid mine leakage, a Yellowstone hot spring and a phosphorus removing sludge community), see Table 1. Our aim was to include metagenomes isolated from diverse extreme environmental sources, where chemical stress is present in addition to thermal effects.

The first discovered hyperthermophilic organism, *Sulfolobus acidocaldarius*, was found in Yellowstone National Park [13]. Nowadays, there are more than 90 known hyperthermophilic species, most of them are archaea, but there are some hyperthermophilic bacteria as well [13].

The metagenomes of two deep-sea hydrothermal vent chimneys, a black-smoker chimney called 4143-1 and a carbonate chimney from Lost City, were investigated in a survey [14]. The samples of these two chimneys are enriched in genes associated with mismatch repair (MMR) and homologous recombination repair [14]. These findings imply that these microorganisms have specific and extensive DNA repair systems to survive under the extreme environmental conditions (such as heavy metals, high concentrations of hydrogen sulfide, radionuclides and high temperature) present in their habitat [14].

(Hyper)thermophilic organisms are exposed to high temperatures, which may be expected to elevate the rate of spontaneous DNA mutations [13]. Interestingly, however, the genomic mutation rate of the hyperthermophilic archaeon *S. acidocaldarius* was found to be equal to that of mesophilic organisms [13]. It was also shown that base substitution rate in *S. acidocaldarius* is 10-fold lower compared to mesophilic organisms [13], [15]. Surprisingly, *S. acidocaldarius* lacks all known bacterial mismatch repair genes. One explanation for this finding may involve a much better proofreading potency and insertion accuracy. Alternatively, a specific novel mismatch repair, distinct from the bacterial mutHLS model, can also account for that “normal” level of replication infidelity even in an extreme environment [15].

The structure of archaeon *Pyrococcus furiosus* proliferating cell nuclear antigen protein (PCNA) is an example of protein adaptation to increased temperature. The reduction of polar uncharged residues and elevated numbers of ion pairs likely contribute to increased stability of PCNA [16]. Besides, archaeal PCNAs are capable of self-loading onto DNA that can help higher DNA repair efficiency at a presumably increased DNA damage rate at extreme conditions [16].

Halophile and acidophile organisms live in relative high concentration of Na^+^ and H^+^, respectively [15]. These microorganisms cannot completely buffer against these ions, which can cause elevated stress to missense mutations carrying organisms. Still, the acidophile *S. acidocaldarius* has a 5-fold lower base substitution rate than the non-acidophile *T. thermophilus* [15], suggesting potent DNA repair systems in action.

The above data indicate that organisms living in extreme environments supposedly suffer more frequent DNA damage than organisms in ambient conditions, and to avoid drastic mutagenesis, they may contain specific and potent DNA repair mechanisms that are more efficient than that of other organisms. Therefore, it may be possible to identify new, more efficient DNA-repair enzymes in these extreme metagenomes. Certainly, there is a remarkable scientific interest in finding novel, more efficient enzymes in exotic species of the metagenomes mentioned. In addition, there is an even stronger interest in finding new functions for already known enzymes and functions for proteins with unknown role in important eukaryotic model organisms, including *Homo sapiens*. Accordingly, we performed a second projection from the DNA-repair enzymes to several model organisms. Application of the Metagenomic Telescope resulted in an increased number of hits, as compared to the one-step original projection. The UniProt [17] accession numbers of these additional hits (“new telescopic hits”), appearing only among the telescopic hits, but not among the original hits are listed in Table S1, together with a short description as found in UniProt.

### HMM projections starting with single domain protein families identify the relevant orthologues with few novel hits

Among the protein families involved in DNA damage recognition and repair selected for this present study, the trimeric dUTPase family — containing five well-conserved characteristic sequence motifs involved in building the active site — constitutes a well defined protein fold which can be also found in the family of prokaryotic dCTP deaminases [18]. In eukaryotes, however, to our present knowledge, this peculiar protein fold is exhibited only by dUTPases and no other proteins. Also, eukaryotic dUTPases are described as monogenic in the model eukaryotic organisms studied to date. dUTPases are responsible for hydrolysis of dUTP thereby preventing uracil incorporation into DNA and generating dUMP, the precursor for dTTP biosynthesis. These enzymes are essential to maintain genome integrity, and are found in all free-living organisms as well as in numerous DNA viruses as well as retroviruses [19]. Although it has been suggested that viral dUTPase sequences encode viral pseudo-proteases [20], later this suggestion was proved to be incorrect [21]; although some database entries still contain the obsolete “pseudo–protease” annotation.

**Figure 3:**
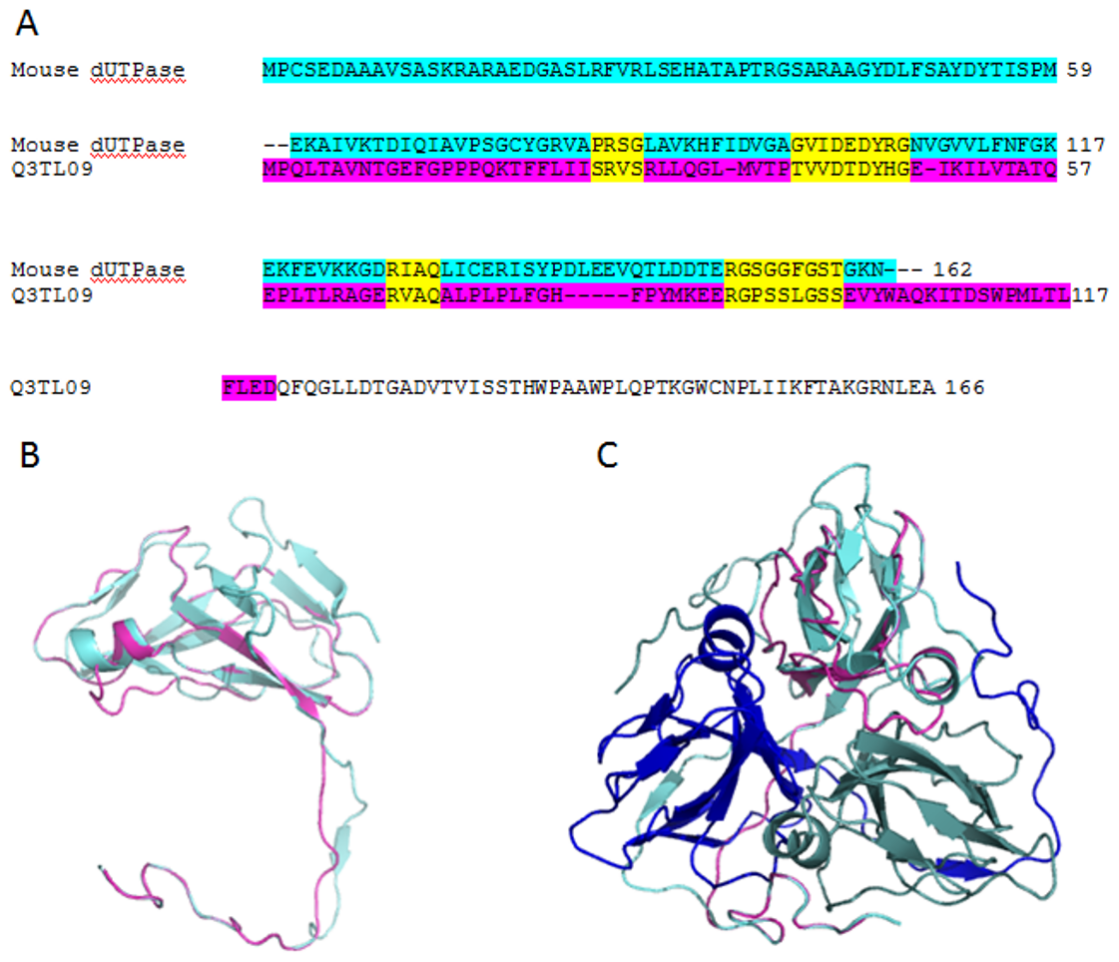
Identification of a novel dUTPase-like protein in the mouse proteome using the Metagenomic Telescope. Panel A shows an alignment (created by ClustalW) between the mouse dUTPase sequence (cyan) and the novel hit associated with the Uniprot accession number Q3TL09 (purple color indicates the part of this latter sequence that could be modeled in 3D using SwissModel or MUSTER). The conserved dUTPase motifs are shown in yellow. Panel B illustrates the structural alignment between human dUTPase (cyan) and the Q3TL09 modeled structure (purple) (at the subunit level). Panel C shows one of the models for Q3TL09 created by MUSTER software (purple), in this case the trimeric structure characteristic of dUTPases is shown (monomers are in shades of blue: cyan, royal blue and grayish blue). Protein structural models are shown in ribbon diagrams (PyMol).

In accordance with the well-conserved character of this protein family, HMM searches did indeed find the orthologous dUTPase sequences, however, no novel protein could be found among the original hits. Still, among the telescopic hits, we found one novel hit in the mouse proteome (UniProt accession number Q3TL09). Although on the sequence level it showed rather low similarity to the authentic dUTPase sequence (identitity 9%, similarity 23%), the sequence alignment indicates that out of the five characteristic dUTPase motifs, four can be identified in the sequence of this protein (Figure 3A). The actual functional relevance of this protein to dUTPases needs further experimental studies out of the scope of the present work. It was also of interest to investigate if the 3D structure of this protein may be similar to the dUTPase fold [22], [23]. For such investigations, first we run the SwissModel software [24], [25] by nominating the human dUTPase 3D structure (PDB ID 3EHW) [26]–[28] as the template. Results showed that the dUTPase fold can be adopted by this protein, however, the strength of this conclusion is somewhat weakened by the fact that the template was pre-defined and could strongly perturb the results.

Hence, we next used the MUSTER software [29] without any pre-defined template. This recently described software is based on an integrated use of protein profiling information and tries to fit a 3D structure from the Protein Data Bank on the sequence submitted. Results of the MUSTER-modeling showed that three slightly different 3D models could be created, and very interestingly, all of these used a dUTPase structure as the best-fitting model (Figures 3B,3C).

We conclude that for the dUTPase searches, the use of the telescopic HMM resulted in a promising finding. The newly found mouse protein, although with a very low level of sequence identity, may adopt the 3D structure of the antiparallel beta-sheeted jelly roll dUTPase-fold.

HMM models were also created for the numerous DNA-glycosylase families (listed in Table 1) that belong to either the alpha/beta superfamily of uracil-DNA glycosylases (UNG, TDG) or to the helix-turn-helix (HTH) superfamily of DNA glycosylases (NTH, NEI, OGG) [30]. These proteins, similarly to dUTPases, are also single domain proteins, with some N- or C-terminal extensions in several eukaryotic organisms. In several cases, eukaryotes encode different isoforms of DNA-glycosylases, dedicated to the different cellular compartments (nuclear vs. cytoplasmic). We found that while the original hits usually included the orthologues and their isoforms, the telescopic hits also included hits from the whole superfamily. For example, starting with the uracil-DNA glycosylase UNG, original hits showed the orthologous nuclear and mitochondrial isoforms of UNG, while telescopic hits included the closely related thymine-DNA glycosylases as well as SMUGs. Similarly, starting from any of the HTH superfamily DNA glycosylases, original hits were rather restricted to the different isoforms of the same proteins, while telescopic hits included proteins of the whole HTH superfamily. Hence, for the cases of the DNA-glycosylase families, the Metagenomic Telescope approach yielded new telescopic hits within the larger superfamily of these repair enzymes, but did not identify proteins within additional new families.

### HMM projections of multi-domain proteins: telescopic hits suggest numerous novel associations

**Figure 4:**
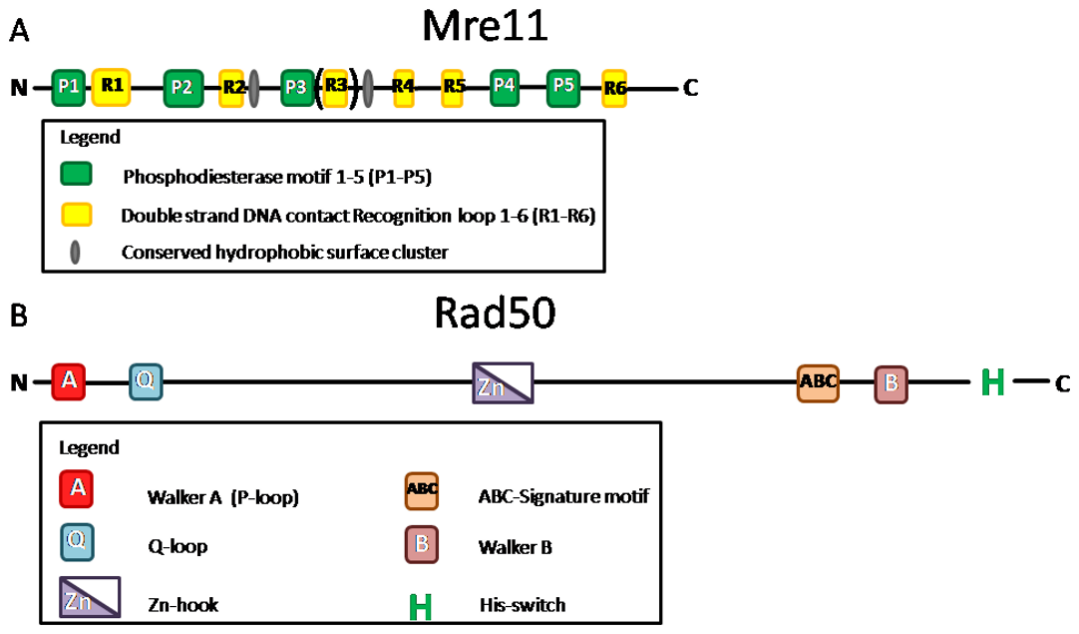
Schematic representation of Mre11 (Panel A) and Rad50 (Panel B) domains. (A) Mre11 has five phosphodiesterase motifs (green), 6 dsDNA recognition loop (yellow) and hydrophobic surface clusters (grey) (B) Rad50 has a bipartite ATPase domain: Walker A (red), Walker B (pale red), Q-loop (light blue), ABC-Signature motif (orange), histidine switch (green H) and has a Zinc-hook (purple).

The Mre11 and Rad50 proteins play important roles in the repair of double-strand-DNA breaks. These two proteins are essential in both major pathways of double-stranded DNA break repair, in homologous recombination repair, as well as in non-homologous end-joining. Both Rad50 and MRE11 are multidomain proteins (cf., Figure 4). Rad50 has an ATPase globular domain and a highly lengthened coiled-coil domain connected together with a Zn-hook, whereas Mre11 contains a phosphodiesterase core domain and several DNA-binding recognition loops.

Rad50 and Mre11 usually form a heterotetramer and this assembly is termed as the MRN complex. The MRN complex is crucial to (i) bridge DNA over short and long distances, (ii) DNA binding and processing, and (iii) activation of double strand break response and checkpoint signaling pathways [31]. Both Mre11 and Rad50 need a metal cofactor: manganese and magnesium, respectively [32]. Both of them can bind DNA. The dimerization of Rad50 is ATP dependent [33] and it belongs to the ABC-ATPase family [33]. Rad50 has a conserved “signature motif” that is needed for binding the *γ*-phosphate of ATP and is characteristic for ABC-ATPases [33]. The “signature motif” has a key role in the Rad50 dimer assembly [33]. Q-loop binds a magnesium ion [33]. The Walker A motif binds ATP and the Walker B motif hydrolyses it. The Walker A motif (also called P loop or phosphate-binding loop) forms the nucleotide binding site [32]. The D loop, which is a part of Walker B, binds one active magnesium ion and assists in dimerization [33]. The Mre11 binding site is on the coiled-coil region adjacent to the ABC domain [32]. Mre11 has five conserved phosphodiesterase motifs [32]. Conserved hydrophobic surface clusters are likely involved in macromolecular interaction sites [32]. The six DNA recognition loops (R1-R6) constitute a continuous DNA interaction surface [34]. All core DNA recognition loops are conserved in *S. pombe, S. cerevisiae and Xenopus*, except recognition loop 3 (R3) [34]. Rad50 and Mre11 homologs in *Escherichia coli* are termed SbcC and SbcD, respectively [35], [ 36].

**Figure 5:**
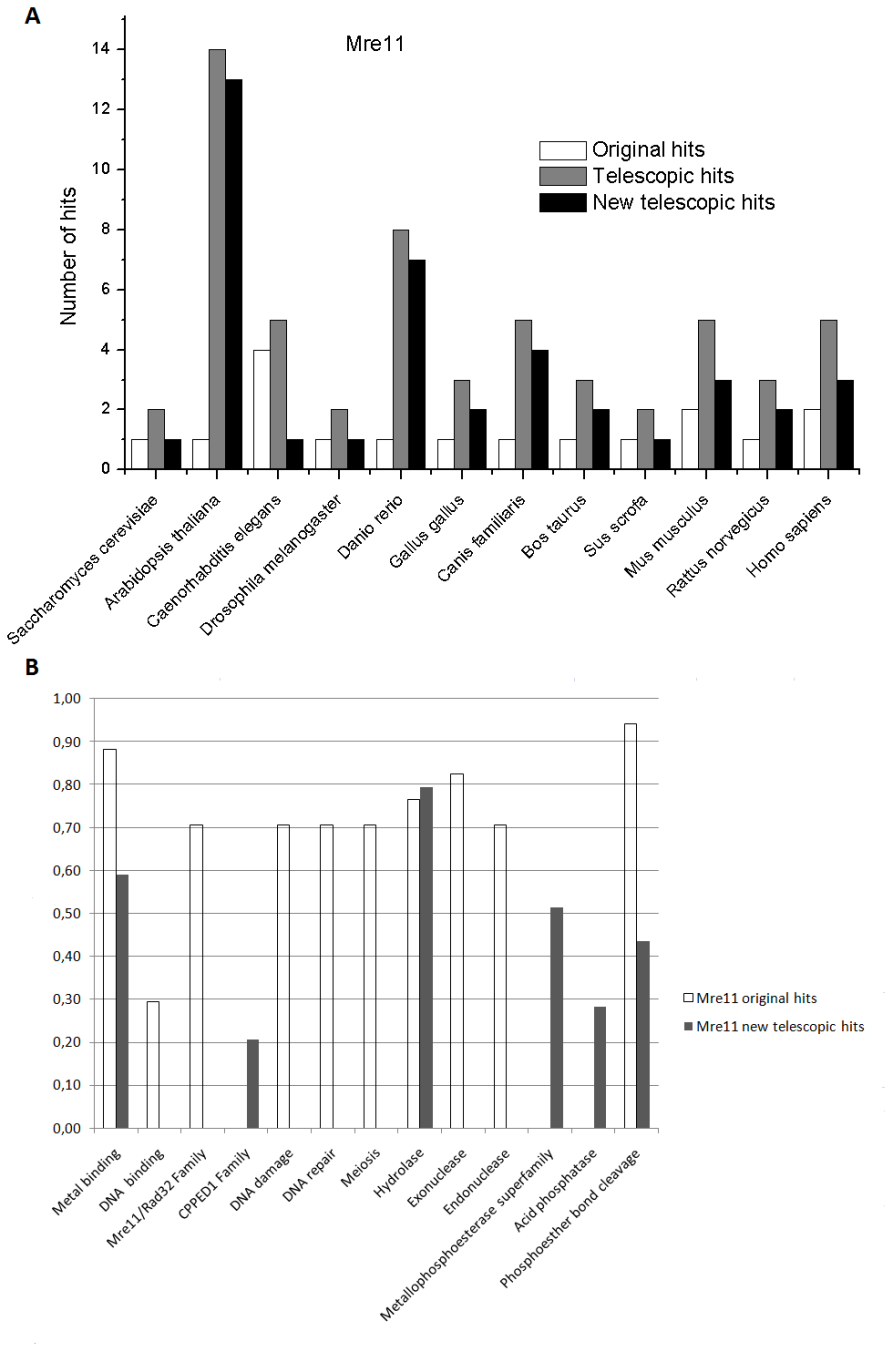
Original and telescopic hits for the Mre11 family. Panel A. Number of hits identified in the various eukaryotic model organisms after the original and the telescopic projections. Panel B. Distribution of genome ontology terms within the different hits. Note that new genome ontology classes can be observed in the telescopic hits.

**Figure 6:**
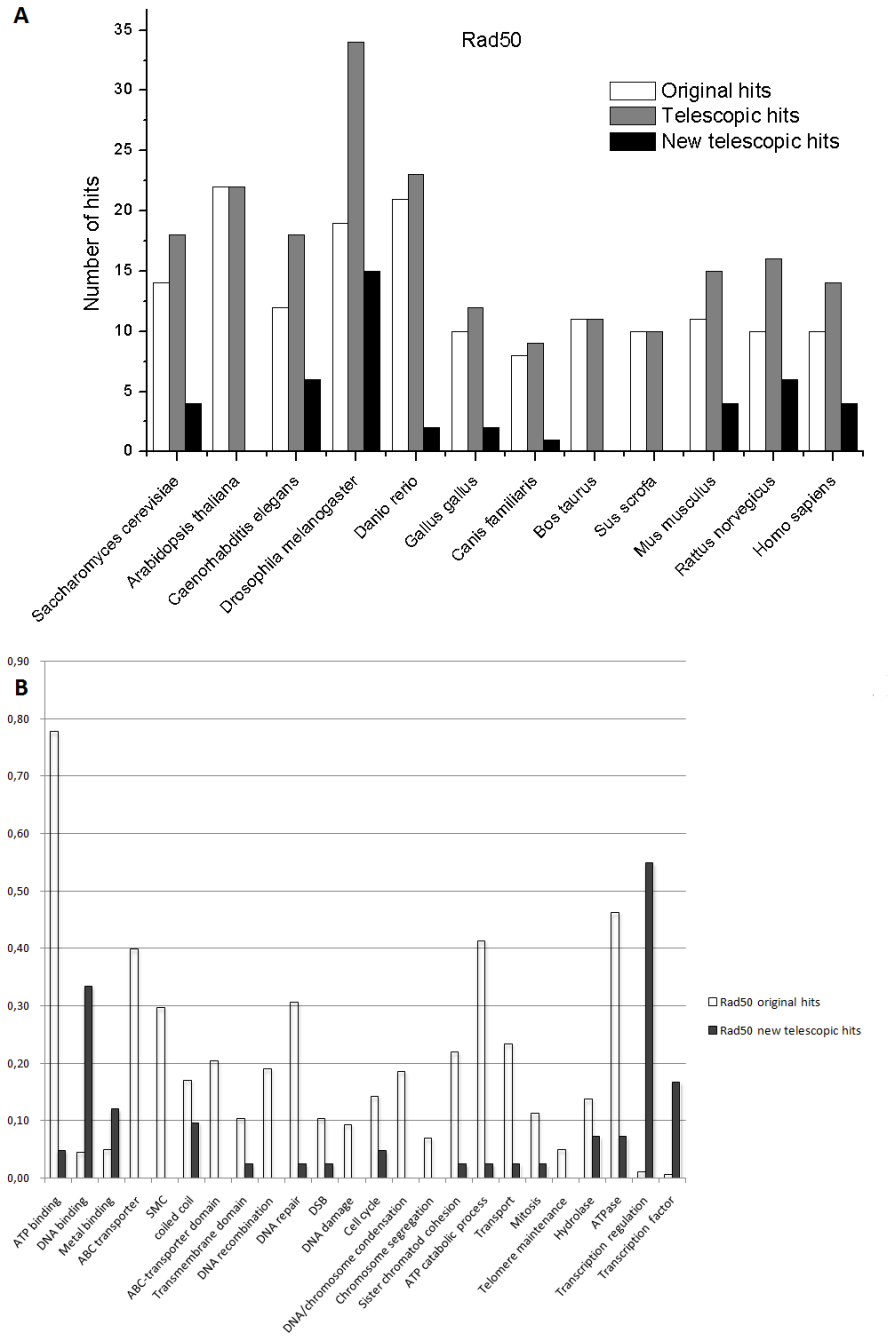
Original and telescopic hits for the Rad50 family. Panel A. Number of hits identified in the various eukaryotic model organisms after the original and the telescopic projections. Panel B. Distribution of genome ontology terms within the different hits. Note that new genome ontology classes can be observed in the telescopic hits.

The results of the application of the Metagenomic Telescope on these protein families are summarized on Figures 5 and 6 (for Mre11 and Rad50, respectively). In both figures, one panel (Figures 5A and 6A) shows the actual number of hits found in the original as well as in the telescopic projections in the model eukaryotic organisms. This representation provides a rather straightforward measure of the strength of the telescopic projection over the original projections. In some cases, the number of hits is just 1 (e.g., in the case for the original hits of Mre11 in several model organisms). In these cases, the hit was actually the *bona fide* Mre11 homologue in the given organism, and no additional “similar” proteins can be found. However, in the majority of cases, the number of hits is more than 1, and in these cases, in addition to the *bona fide* homologue that was always among the hits, additional proteins were also identified by the HMM projections.

The fact that the *bona fide* homologue is always identified indicates that the HMM projections are reliable. Nevertheless, these are the additional hits that may contain novel properties. It is easy to see for both Mre11 and Rad50 that the number of hits for a telescopic projection is never smaller than that for the corresponding original projection, on the contrary, these hits are quite frequently significantly more numerous. The additional hits, identified only in the telescopic projections are termed “new telescopic hits” on the respective panels in Figures 5A and 6A (cf. also Table S1).

To analyze the putative biological functions of the original and the new telescopic hits, in each cases we relied on the genome ontology classification categories, as provided in the UniProt database, and listed the different genome ontology definitions for each hit. The biological functions (genome ontology categories) found to be associated with most of the original hits are rather straightforward to assess. Accordingly, for both the Mre11 and Rad50 families, we find that the functions listed (metal binding, DNA binding, DNA repair, etc) are already known to be associated with the Mre11 and Rad50 families.

Next, we compared the original and telescopic hits and found that the list of these properties is significantly enriched in the telescopic hits. Therefore, not only the number of hits was higher after using the telescopic HMMs, but also these hits were associated with additional functional properties (Figures 5B and 6B). The new telescopic hits were identified starting from different protein families involved in DNA repair. The criteria to affirm if any of these hits belong to e.g., families of Rad50 or Mre11 was to observe if these hits are listed in the UniProt database as belonging to the given protein family.

In order to evaluate the power of the Metagenomic Telescope method, we need to consider those genome ontology terms that show up only in the new telescopic hits. For the Mre11 family, such terms are the calcineurin-like phosphoesterase (CPPED1) family, the metallophosphoesterase family and the acid phosphatase biological function. While the latter two may be explained by the well-known characteristics of the Mre11 enzymatic action, the connection to the calcineurin-like phosphoesterase family seems to be novel. In this case, at least to our knowledge, the potentially similar characteristics of Mre11 and calcineurin-like phosphoesterases have not yet been addressed before. In the case of the hits within the Rad50 family, the novel hits using the telescopic projections are even more evident. Perhaps the most intriguing result from these projections concerns the numerous occurrence of the “transcription regulation” and “transcription factor” genome ontology classes, which are evidently linked.

Table 2 presents these new telescopic hits, where we also listed the actual proteome within which the hits were identified. It is evident that these hits belong the different families involved in transcription regulation, each associated with its characteristic sequence motifs. Based on these findings, we suggest that Rad50-like proteins may also be involved not just in interacting with DNA but also interacting with the transcription process. It is known that e.g., DNA damage and repair occurs with higher frequency on transcriptionally active genomic segments since these are more accessible. Our present results may suggest that in addition to the less physical barrier in the actively transcribed genomic regions, Rad50-like proteins may also be involved in interacting with the transcription machinery in a more direct manner.

**Table 2:** New telescopic hits associated with a role in transcription regulation.

UniProt	Description	Organism
P18480	SWI/SNF chromatin-remodeling complex subunit SNF5	S.cer.
P29617	Homeobox protein prospero (May regulate transcription by binding to DNA)	D.mel.
P21519	Neurogenic protein mastermind	D.mel.
Q9VZY2	Myocardin-related transcription factor	D.mel.
Q24167	Protein similar (transcriptional regulator of the adaptive response to hypoxia)	D.mel.
Q9VPL6	Kismet (Hydrolase)	D.mel.
A8JNQ5	Ataxin-2 binding protein 1	D.mel.
Q8IQ98	PAR-domain protein 1	D.mel.
Q9VSK5	Grunge (Hydrolase)	D.mel.
P13002	Protein grainyhead/DNA-binding protein ELF-1/Transcription factor NTF-1	D.mel.
F1NZW0	Transcription initiation factor TFIID subunit 3 (Zinc-finger domain)	G.gal.
F1NY57	Uncharacterized protein (Contains: fork-head DNA-binding domain)	G.gal.
G3X8S4	Mediator of RNA polymerase II transcription subunit 15	M.mus.
Q8K4J6	Myocardin-like protein 1/Basic SAP coiled-coil transcription activator/	
	Myocardin-related transcription factor A	M.mus.
D4QGC2	Mastermind-2 (transcription coactivator activity -positive regulation of	
	transcription from RNA polymerase II promoter)	M.mus.
G3V684	Positive cofactor 2/multiprotein complex/glutamine/Q-rich-associated	
	protein (role in: stem cell maintenance)	R.nor.
F1LV40	Protein Mkl1 (negative regulation of cysteine-type endopeptidase activity	
	involved in apoptotic process)	R.nor.
F1M7D7	Forkhead box protein P2	R.nor.
O14686	Histone-lysine N-methyltransferase 2D	Human
Q8IZL2	Mastermind-like protein 2	Human
Q0PRL4	Forkhead box P2	Human
Q96RN5	Mediator of RNA polymerase II transcription	Human

Using Rad50 sequences to build the starting HMM model, the Metagenomic Telescope approach identified the new telescopic hits listed in the table.

Our present method applied to the Mre11 and Rad50 protein families identified several new telescopic hits that are predicted to possess functional properties originally not found in Mre11 or Rad50. These proteins, according to the UniProt database, belong to protein families that still show some common traits with the Mre11 or Rad50 families with respect to catalytic action on nucleoside phosphates and/or nucleic acid binding. However, these newly found telescopic hits are not described in the UniProt database as members of the Mre11 or Rad50 families. We conclude that using the information within the metagenomes, the Metagenomic Telescope method leads to protein hits outside the protein families originally used as starting sequences, potentially facilitating search for proteins with additional functions.

## Supporting Information

Table S1 **New telescopic hits identified in the present study.**
(XLSX)
